# Coffee Silver Skin: Chemical Characterization with Special Consideration of Dietary Fiber and Heat-Induced Contaminants

**DOI:** 10.3390/foods10081705

**Published:** 2021-07-23

**Authors:** Vera Gottstein, Mara Bernhardt, Elena Dilger, Judith Keller, Carmen M. Breitling-Utzmann, Steffen Schwarz, Thomas Kuballa, Dirk W. Lachenmeier, Mirko Bunzel

**Affiliations:** 1 Chemisches und Veterinäruntersuchungsamt (CVUA) Karlsruhe, Weissenburger Straße 3, 76187 Karlsruhe, Germany; Vera.Gottstein@cvuaka.bwl.de (V.G.); Elena.Dilger@cvuaka.bwl.de (E.D.); Thomas.Kuballa@cvuaka.bwl.de (T.K.); Lachenmeier@web.de (D.W.L.); 2 Department of Food Chemistry and Phytochemistry, Karlsruhe Institute of Technology (KIT), Adenauerring 20A, 76131 Karlsruhe, Germany; mara-s.bernhardt@gmx.de (M.B.); judith.schaefer@kit.edu (J.K.); 3 Chemisches und Veterinäruntersuchungsamt Stuttgart, Schaflandstr. 3/2, 70736 Fellbach, Germany; Carmen.Breitling-Utzmann@cvuas.bwl.de; 4 Coffee Consulate, Hans-Thoma-Stasse 20, 68163 Mannheim, Germany; schwarz@coffee-consulate.com

**Keywords:** coffee silver skin, dietary fiber, elements, heat-induced contaminants

## Abstract

Coffee silver skin is produced in large amounts as a by-product during the coffee roasting process. In this study, coffee silver skin of the species *Coffea arabica* L. and *Coffea canephora* Pierre ex A. Froehner as well as silver skin pellets produced in the coffee industry were characterized with respect to both nutritional value and potential heat-induced contaminants. Enzymatic-gravimetric/chromatographic determination of the dietary fiber content showed values ranging from 59 to 67 g/100 g with a comparably high portion of soluble fiber, whereas low molecular weight soluble fiber was not detected. Compositional and methylation analysis indicated the presence of cellulose and xylans in the insoluble dietary fiber fraction, whereas pectic polysaccharides dominate the soluble dietary fiber fraction. The protein content as determined by the Kjeldahl method was in the range of 18 to 22 g/100 g, and all essential amino acids were present in coffee silver skin; whereas fat contents were low, high ash contents were determined. Elemental analysis by inductively coupled plasma mass spectrometry (ICP-MS) showed the presence of macroelements in large amounts, whereas toxic mineral elements were only detected in trace amounts or being absent. Acrylamide was quantified with levels of 24–161 µg/kg. Although 5-hydroxymethylfurfural was detected, its concentration was below the limit of determination. Furfuryl alcohol was not detected.

## 1. Introduction

**Figure 1:**
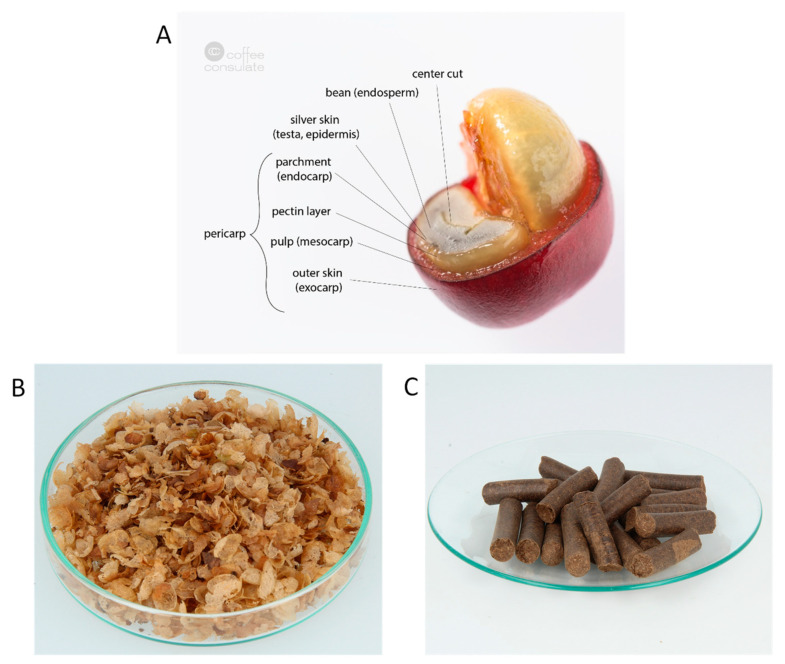
Profile of a coffee cherry with its various layers (**A**) Reprinted from Ref. [5], photo of coffee silver skin (**B**) Reprinted from Ref. [5], and photo of coffee silver skin pellets (**C**).

Coffee is a popular beverage all over the world, consumed mainly for its stimulating properties. Economically important are the two species *Coffea arabica* L. (*arabica*) and *Coffea canephora* Pierre ex A. Froehner (*canephora*), with *arabica* fetching a higher price in the market due to its organoleptic properties, which are considered to be of higher quality [1]. Coffee is commonly consumed as an infusion, which is prepared from roasted coffee beans [2]. To obtain roasted coffee beans, several steps are necessary in which different coffee by-products are generated [3,4]. One of these by-products is coffee silver skins (CS) (Figure 1B), which partially remain on the green beans after processing of the coffee cherries [5,6]. CS are a thin tegument (Figure 1A) that builds the outer layer of the green coffee bean and represents 4.2% (*w*/*w*) of the coffee cherry [6].

CS are separated from the beans and accrue as a by-product of the roasting process, which typically no longer takes place in the growing countries. In some countries, CS are used as a fuel, but beyond that, efficient use of CS especially in the food industry is not described [6,7]. However, sporadic studies report potential applications of CS. For example, Pourfarzad et al. described CS as an ingredient in barbari flat bread [8], whereas Garcia-Serna et al. investigated the addition of stevia and CS to biscuit dough [9]. Bertolino et al. studied the addition of CS to yogurt in order to enrich its contents in dietary fiber, phenolic compounds, and caffeine [10]. Thus, the application of CS in the food industry appears to be attractive due to the composition of these products described so far [3,9,11,12,13,14,15]. In addition, aroma compounds produced by the roasting process add a smoke flavor in other food applications [5]. Additionally, Rodrigues et al. and Bessada et al. investigated coffee silver skin as a potential ingredient for cosmetics [11,16]. 

Because CS are derived from coffee beans, their composition is expected to be similar to coffee beans [12]. Thus, beneficial traits of CS, such as a high fiber content of 60–80%, have already been described [3,12,13,14,15]. Dietary fiber is associated with several health benefits, and first attempts to characterize CS dietary fiber composition suggest a possible use of CS as functional ingredient for fiber enrichment of foods [13,17]. Nevertheless, an in-depth characterization of CS fiber structures including detailed information on polysaccharide interunit linkages and lignin was not found in recent literature. In addition, CS have a high antioxidant activity, which can be attributed to the presence of both (poly)phenolic compounds and melanoidins [13,18,19,20]. Some studies did not only describe a high antioxidant activity, but also identified (poly)phenolic compounds such as chlorogenic acids as main contributors to that function [15,18,20]. Tores de la Cruz et al. determined melanoidins in CS, which also showed antioxidant properties [19]. Jiménez-Zamora et al. defined the content of melanoidins in CS to be 15–25% [21]. 

Ingredients of concern, such as mycotoxins, 5-hydroxymethylfurfural (HMF), and acrylamide, were also determined [3,22]. A contamination with mycotoxins, specifically ochratoxin A (OTA), in amounts ranging from 18 to 36 µg/kg, was noted by Toschi et al. [23]. OTA has been classified as possibly carcinogenic to humans (group 2B) by the International Agency for Research and Cancer (IARC) [24]. However, Iriondo-DeHond et al. investigated the acute toxicity of an aqueous extract of CS in rats and observed no visible signs of toxicity or abnormal behavior [3]. 

In order to use CS in the food industry, both a comprehensive characterization of the major CS components and an analysis of heat-induced contaminants is still required. These include compounds that can be formed during roasting, such as acrylamide, furfuryl alcohol, or HMF, some of which have been classified as possible (furfuryl alcohol) or probable (acrylamide) human carcinogens by the IARC [25,26]. Although some studies already described heat-induced contaminants such as HMF or acrylamide in CS [3,9,22,27,28], data on furfuryl alcohol have, to the best of our knowledge, not been published yet. CS from the different species *Coffea arabica* and *Coffea canephora* from different geographical origins, as well as mixtures thereof, have already been investigated [3,18,19,20,22,29]; our study does not only include CS from *Coffea arabica* and *Coffea canephora*, but CS pellets (Figure 1C) produced in the coffee industry were investigated for their composition, too.

## 2. Materials and Methods 

### 2.1. Materials

CS from the species *Coffea arabica* (*arabica* CS) and *Coffea canephora* (*canephora* CS) as well as CS pellets were provided by Coffee Consulate (Mannheim, Germany). *Canephora* CS originated from India, where coffee cherries were processed mainly wet (fully washed). *Arabica* CS were from Brazil, India, Mexico, and El Salvador, where coffee cherries were processed both, dry and wet. The roasting process to separate CS took place in a Ghibli R15 commercial roaster (Coffee-Tech Engineering, Moshav Mazliach, Israel). CS from different roasting batches were collected over approximately 2–3 months. After roasting, all CS were stored at room temperature until further processing. CS pellets were produced by pressing CS into a pellet mold. Because the pellets were produced in large roasting facilities, it can be assumed that they are composed of approximately 70% *Coffea arabica* and 30% *Coffea canephora* CS, which corresponds to the typical coffee blend commercially available in Germany. CS were ground to a particle size <0.5 mm using a ZM 200 ultra centrifugal mill (Retsch, Haan, Germany) and stored at room temperature until analysis. 

### 2.2. Chemicals

All reagents and standard compounds used were of analytical or high performance liquid chromatography (HPLC) grade. Acetone, acetonitrile, chloroform, sodium dihydrogen phosphate dihydrate, petroleum ether (40–60 °C), and sulfuric acid were from VWR (Bruchsal, Germany). Acetyl bromide, *N*,*O*-bis(trimethylsilyl)trifluoroacetamide (BSTFA) with 1% TMCS, bisphenol, *m*-hydroxybiphenyl, methanolic hydrochloric acid (1.25 M), sodium borohydride, sodium tetraborate, sodium hydroxide, trifluoroacetic acid, Trizma base, 1-methylimidazole, and Driselase from *Basidiomycetes* sp. were obtained from Sigma Aldrich (Darmstadt, Germany). Chloroform-*d*_1_, celite, sodium borodeuteride, *o*-phosphoric acid, and *tert*-butyl methyl ether were from Carl Roth (Karlsruhe, Germany). Ammonia, ammonium chloride, acetic acid, acetic anhydride, magnesium oxide, methanol, nitric acid (65%), and hydrogen peroxide (30%) were from Merck (Darmstadt, Germany). Kjeldahl catalyst and sodium dihydrogen phosphate were from Fluka (Munich, Germany), disodium hydrogen phosphate was from Riedel-de Haën (Seelze, Germany), ethanol was from Brüggemann (Heilbronn, Germany), methanolic trimethyl sulfonium hydroxide solution (0.2 M) was from Macherey-Nagel (Dueren, Germany), and 6-aminoquinoline-*N*-hydroxy-succinimidyl-carbamate (AQC) was purchased from Chemodex (Hamburg, Germany).

Thermally stable α-amylase (Termamyl 120 L) from *Bacillus licheniformis*, amyloglucosidase (AMG 300 L) from *Aspergillus niger*, and protease (Alcalase 2.5 MG Type FG) from *Bacillus licheniformis* were from Novozymes (Bagsvaerd, Denmark). α-Amylase from porcine pancreas, amyloglucosidase from *Aspergillus niger*, protease from *Bacillus licheniformis* were from Megazyme (Bray, Ireland).

ICP multi-element standard solution VI with 30 elements (Ag, Al, As, B, Ba, Be, Bi, Ca, Cd, Co, Cr, Cu, Fe, Ga, K, Li, Mg, Mn, Mo, Na, Ni, Pb, Rb, Se, Sr, Te, Tl, U, V, Zn) diluted in nitric acid (1%) was from Merck (Darmstadt, Germany).

### 2.3. Methods

#### 2.3.1. Insoluble, Soluble, and Low Molecular Weight Soluble Dietary Fiber 

Insoluble dietary fiber (IDF), soluble dietary fiber (SDF), and low molecular weight soluble dietary fiber (LMSDF) were determined using the enzymatic gravimetric/chromatographic method according to McCleary et al. [30]. LMSDF was estimated by using high performance liquid chromatography in combination with a refractive index detector (HPLC-RI, Hitachi, Düsseldorf, Germany). Parameters for HPLC-RI analysis were as follows: column, TSKgel G2500PWxl (3000 mm × 7.8 mm, Tosoh, Grießheim, Germany), 2× in series; eluent, double distilled water with a constant flow rate of 0.4 mL/min; oven temperature, 80 °C. Total dietary fiber (TDF) was calculated from IDF, SDF, and LMSDF. To structurally characterize IDF and SDF, a preparative isolation procedure of dietary fiber was performed (resulting in preparative IDF/SDF) using, among others, a thermostable α-amylase instead of a pancreatic α-amylase as detailed in [31]. Because analyses of IDF, SDF, and LMSDF were carried out in duplicate, range/2 was reported as a suitable measure to indicate statistical dispersion.

##### Determination of the Monosaccharide Composition of Insoluble and Soluble Dietary Fiber

The neutral monosaccharide composition of preparative IDF was determined after sulfuric acid hydrolysis according to Saeman et al. [32], followed by gas chromatographic analysis coupled to a flame ionization detector (GC-FID). Sulfuric acid (1.5 mL, 12 M) was added to 100 mg of IDF. The suspension was placed in an ice bath for 30 min, followed by a treatment at room temperature for 2 h. The suspension was diluted with 9.75 mL of water, and IDF polysaccharides were hydrolyzed for 3 h at 100 °C. After filtering through a membrane filter (0.45 µM, PTFE), 250 µL of the filtrate was used for derivatization. The filtrate was mixed with 90 µL of ammonia (0.1 M) and 660 µL of water. An aliquot of 100 µL was incubated with 1 mL of sodium borohydride solution (2%) at 60 °C for 1 h. After cooling down to room temperature, 70 µL of glacial acetic acid and 20 µL of erythritol + inositol solution (25 mM), 2 mL of acetic anhydride, and 200 µL of the catalyst 1-methylimidazole were added. After 10 min, 5 mL of water was added under ice cooling. Formed alditol acetates were extracted into 2 mL of chloroform, which was washed twice with 2 mL of water. After residual water was frozen out, the chloroform phase was used for GC-FID (GC-2010 Plus, Shimadzu, Germany) analysis on an HP5 capillary column (30 m × 0.35 mm, i.d., 0.25 µm film thickness, Agilent Technologies, Waldbronn, Germany). The following temperature program was used: initially 150 °C (held for 3 min), ramped at 5 °C/min to 200 °C (held for 5 min), 2.5 °C/min to 220 °C (held for 5 min), and finally, 20 °C/min to 300 °C (held for 10 min). Split injection with a split ratio of 1:6 was used. The injection volume was 1 µL, and injection temperature was set to 250 °C. FID temperature was 300 °C. Hydrogen was used as carrier gas, nitrogen as makeup gas. Neutral monosaccharides were expressed as a percentage after semi-quantitative determination of the corresponding alditol acetates using previously determined response factors. Response factors were determined from an aqueous mixture of apiose, rhamnose, fructose, arabinose, xylose, mannose, glucose, and galactose (3 mM each). An aliquot of the standard solution (100 µL) was evaporated, redissolved in 100 µL of 0.1 M ammonia solution, and derivatized and analyzed analogously to the sample. Determination of monosaccharide composition was carried out in duplicate; range/2 indicates the statistical dispersion within this semiquantitative estimation of the composition.

The monosaccharide composition of preparative SDF was determined by methanolysis as described by De Ruiter et al. [33] with minor modifications according to Wefers & Bunzel [34] (methanolysis using 1.25 M HCl in methanol, 80 °C, 16 h; followed by hydrolysis using 2 M trifluoroacetic acid, 121 °C, 1 h). Dried residue after methanolysis was dissolved in 100 µL of 0.1 M ammonia solution. Derivatization and GC-FID analysis were performed as described above. 

Uronic acid contents of IDF and SDF were determined spectrophotometrically according to the method of Blumenkrantz and Aboe-Hansen [35]. IDF or SDF (10 mg) were mixed with glass beads (1.23–1.55 mm) and 300 µL of 12 M sulfuric acid. Samples were left in an ice bath for 30 min (shaking every 5 min) and stored at room temperature for 2 h (shaking every 10 min). After the addition of 1950 µL of water, samples were membrane filtrated (0.45 µm, PTFE) and diluted (IDF 1:10, SDF 1:50). Under cooling in an ice bath, sulfuric acid sodium tetraborate (3.6 mL, 0.0125 M in 18 M H_2_SO_4_) was added to 600 µL of the sample solution. Incubation was carried out at 100 °C for 5 min, followed by cooling in an ice bath. The solution was mixed with 60 µL of *m*-hydroxydiphenly solution (0.15% in 0.5% sodium hydroxide solution) and immediately measured by using an UV/VIS spectrophotometer (V-550, Jasco, Pfungstadt, Germany). Calibration was performed using galacturonic acid (10, 25, 40, 60, 85, 100 mg/L), and water served as blank. Uronic acid contents were not included in the (neutral) monosaccharide composition because different methods were used. Measurement of uronic acid content was performed in duplicate, too, and data are indicated as mean ± range/2.

##### Methylation Analysis of Insoluble and Soluble Dietary Fiber Polysaccharides

Permethylation of IDF and SDF polysaccharides was carried out according to the principle published by Ciucanu and Kerek [36] (methylation with methyl iodide in DMSO/NaOH), with methylation of IDF being performed twice. The methylation analysis protocol was performed according to Gniechwitz et al. [37] and Wefers & Bunzel [34] with the following minor modifications: After dissolving/suspending IDF and SDF (5 mg) in 2 mL of dimethyl sulfoxide, the suspension/solution was sonicated for 15 min, allowed to swell overnight, and sonicated again for 15 min before sodium hydroxide (100 mg) was added. Analysis of partially methylated alditol acetates (PMAAs) by gas chromatographic analysis coupled to mass spectrometry (GC-MS, identification) and GC-FID (semiquantitative analysis using the concept of molar response factors according to Sweet et al. [38]) was performed as published by Wefers & Bunzel [34]. Methylation analysis was performed in duplicate (mean ± range/2).

#### 2.3.2. Determination of Lignin Contents, Characterization of Lignin Monomers

Lignin contents were analyzed as both Klason lignin and as acetyl bromide soluble lignin (ABSL) as described by Schäfer et al. [39]. Klason lignin was determined gravimetrically after a two-step sulfuric acid hydrolysis. Klason lignin contents were corrected for residual protein and ash. Prior to the actual lignin determination as ABSL, polysaccharides were partially degraded using the enzyme preparation Driselase in order to enrich lignin and to minimize lignin overestimation based on carbohydrate degradation products. Following derivatization in 25% acetyl bromide solution in acetic acid, dilution steps using acetic acid, addition of NaOH and hydroxylamine hydrochloride solution, ABSL was determined measuring the absorbance at 280 nm and using the absorption coefficient according to Iiyama et al. [40]. Klason lignin and ABSL were analyzed in duplicate (mean ± range/2). 

Determination of lignin monomers by thioacidolysis was performed according to Yue et al. [41] with minor modifications. Ethyl mercaptan solution (3 mL) and 100 µL of bisphenol E stock solution (2 mM) were added to 10 mg of IDF. The mixture was stirred at 100 °C for 4 h before being cooled in an ice bath for 10 min. After adjusting the pH to 3–4 with 0.4 M NaHCO_3_ solution (0.4 M), 2 mL of water was added. After CHCl_3_ extraction (4 mL, 3×), the combined organic phases were dried over anhydrous MgSO_4_, filtered through a pleated filter, and evaporated under reduced pressure. Residues were dissolved in 1 mL of CH_2_CL_2_, and 10 µL of the solution was silylated (50 µL BSTFA, 40 µL pyridine) at 40 °C for 30 min for GC-MS analysis (GC-2010 Plus and GCMS-QP 2010, Shimadzu, Buchholz, Germany) on a DB-5 column. The temperature program started at 140 °C, and the temperature was ramped at 5 °C/min to 300 °C and held for 10 min. Split injection with a split ratio of 30:1 was used. The injection volume was 1 µL, the injection temperature was set to 220 °C, and helium was used as carrier gas (1 mL/min). Quantification was carried out in the selected ion monitoring (SIM) mode using the following ions: *m*/*z* 239 (*p*-coumaryl alcohol derivative), *m*/*z* 269 (coniferyl alcohol derivative), *m*/*z* 299 (sinapyl alcohol derivative). This semiquantitative approach to characterize lignin monomers was carried out in duplicate (mean ± range/2).

#### 2.3.3. Determination of Protein and Amino Acid Composition

The protein content was determined in duplicate after Kjeldahl digestion using an ammonia selective electrode according to Urbat et al. [42]. Protein content was calculated by external calibration (calibration points: 0.1/1/10 mg N/L). After hydrolysis of CS proteins with hydrochloric acid and derivatization of the liberated amino acids with AQC, the amino acid composition was determined by HPLC with fluorescence detection as detailed in [42]. Determination of amino acid composition was performed in triplicate (mean ± standard deviation). 

#### 2.3.4. Determination of Fat and Fatty Acid Composition

Fat was determined in duplicate by using the German reference method according to Weibull-Stoldt. In brief, CS (5 g) were mixed with 100 mL of water and 100 mL of hydrochloric acid, followed by boiling for 1 h. After filtering through a pleated filter, the filtrate was washed acid-free with water and dried (100 °C, 3 h). Subsequently, the fat was extracted with petroleum ether in an automatic Soxtherm extraction unit (C. Gerhardt GmbH & Co. KG, Königswinter, Germany). After extraction, the solvent was removed, and the residue was weighed (duplicate analyses, mean ± range/2).

To analyze the fatty acid composition, *tert.*-butyl methyl ether (5 mL) was added to the extracted fat (100 mg). An aliquot of 100 µL was mixed with 50 µL of methanol and trimethyl sulfonium hydroxide solution (0.2 M). The derivatized sample was diluted 1:2 with *tert.*-butyl methyl ether and measured by GC-FID (GC-2010 Plus, Shimadzu, Buchholz, Germany) on a DB-5 column (Agilent Technologies, Germany). The temperature program started at 140 °C (held for 1 min) and was ramped at 20 °C/min to 220 °C (held for 25 min). Injection was splitless, injection volume was 1 µL, and injection temperature was 220 °C. Helium was used as carrier gas (1 mL/min), FID temperature was 300 °C. The proportions of the individual fatty acids were determined in relation to the total fatty acid content. Determination of fatty acid composition was carried out in duplicate; these compositional data are indicated as mean ± range/2.

#### 2.3.5. Determination of Ash and Element Composition

Ash contents were determined by incinerating 2.5 g of CS for 5 h at 500 °C. After cooling in the desiccator, the residue was determined gravimetrically. Analyses were performed in duplicate (mean ± range/2).

For acid digestion and mineralization, 500 mg of CS was weighed in quartz vessels and suspended in 1 mL of double-distilled water. After addition of 4 mL of nitric acid (HNO_3_ 65%, suprapur) and 2 mL of hydrogen peroxide (30%, suprapur), the mixture was allowed to stand overnight. Microwave digestion (MultiwavePro, AntonPaar GmbH, Graz, Austria) was carried out at a temperature of 200–210 °C and a pressure of 80 bar. The digested CS solution was diluted to a total volume of 20 mL. Measurements were performed using an ELAN DRC-E inductively coupled plasma mass spectrometer (ICP-MS, PerkinElmer LAS GmbH, Rodgau, Germany) operating in semiquantitative mode for the elemental screening. We used a single calibration standard containing 30 elements (ICP multi-element standard solution VI) in known concentrations and a blank consisting of 1% HNO_3_. The software returns the intensities for each of the calibration elements and calculates the response for a set of elements across the mass range based on the specified concentrations. Elements that are not covered by the standard solution are determined by interpolating between calibrated isotopes. A rhodium solution (10 mg/L) was used as internal standard. All samples were measured in multiple dilutions from at least two independent preparations (*arabica* CS n = 4; *canephora* CS n = 3; CS pellets n = 2). To check the accuracy, a reference material was also measured in each measurement series. The detection limit of this method is exceeded as soon as the intensity of a peak value is higher than 1000. Only measurements with a signal of at least 1E4 to 1E6 cps were used for the evaluation. Measurements with signals above 1E6 cps are subject to errors and annotated in the results; these values should only be used for orientation. 

#### 2.3.6. Determination of Acrylamide, Furfuryl Alcohol, 5-Hydroxymethylfurfural, and Caffeine

Acrylamide was analyzed using liquid chromatography in combination with tandem mass spectrometry (LC-MS/MS) according to the European Standard DIN EN 16618:2015, as described previously [43]. 

Furfuryl alcohol and 5-hydroxymethylfurfural (HMF) were determined in duplicate using nuclear magnetic resonance spectroscopy (NMR) as described elsewhere [44]. Validation parameters for these analytes are described in Table 1 [44]. 

Caffeine was analyzed according to the German reference methodology for the determination of caffeine in coffee and coffee products [45]. In brief, CS (1 g) were mixed with 5 g of magnesium oxide and 100 mL of water and heated at 90 °C for 20 min with constant shaking. After tempering, the solution was made up to 100 mL, filtered through a membrane filter (0.45 µm, PTFE), and analyzed by HPLC coupled with a diode array detector (HPLC-DAD, Agilent, Waldbronn, Germany). Determination of caffeine was carried out in triplicate (mean ± standard deviation). 

#### 2.3.7. Determination of Moisture Content

Moisture content was determined using the automatically operating halogen moisture analyzer HC 103/01 (Mettler Toledo, Gießen, Germany). In brief, 4.5 g of CS were weighed in the moisture meter. Drying was started, and the drying temperature was 105 °C. The evaluation was performed automatically within the instrument, and the instrument displayed the moisture content of the CS after drying. Determination of moisture content was performed in triplicate (mean ± standard deviation).

## 3. Results and Discussion

### 3.1. Total, Insoluble, Soluble, and Low Molecular Weight Soluble Dietary Fiber Contents

TDF contents ranged from 59.1 ± 0.02 g/100 g (*arabica* CS) to 67.0 ± 1.0 g/100 g (pellets) (Table 2), being consistent with TDF values of 50–80 g/100 g previously reported in the literature; however, despite the values cannot directly be compared due to slightly different sample preparation procedures [3,12,13,14,15]. These studies, though, mostly analyzed mixtures of *arabica* and *canephora* CS. Iriondo-DeHond et al. studied *arabica* and *canephora* CS separately and reported TDF contents of 67.7 g/100 g for *arabica* CS and 69.3 g/100 g for *canephora* CS [3]. Nevertheless, Bessada et al. reported TDF contents of *canephora* CS from different countries ranging from 52.9 to 59.6 g/100 g [22]. With concentrations of 56.0 ± 0.4 g/100 g (*arabica* CS), 53.2 ± 0.23 g/100 g (*canephora* CS), and 46.0 ± 0.2 g/100 g (CS pellets), the portion of IDF was higher as compared to SDF (11.0 ± 1.67 g/100 g; 8.8 ± 0.5 g/100 g; 13.01 ± 0.2 g/100 g, Table 2). Thus, pellets show the lowest IDF but highest SDF contents among CS samples. LMSDF was not detected in any of the CS studied. Generally, CS have high levels of dietary fiber including SDF and may therefore be suitable for use as a natural ingredient in functional foods. 

#### 3.1.1. Monosaccharide Composition of Insoluble and Soluble Dietary Fiber Polysaccharides

**Figure 2:**
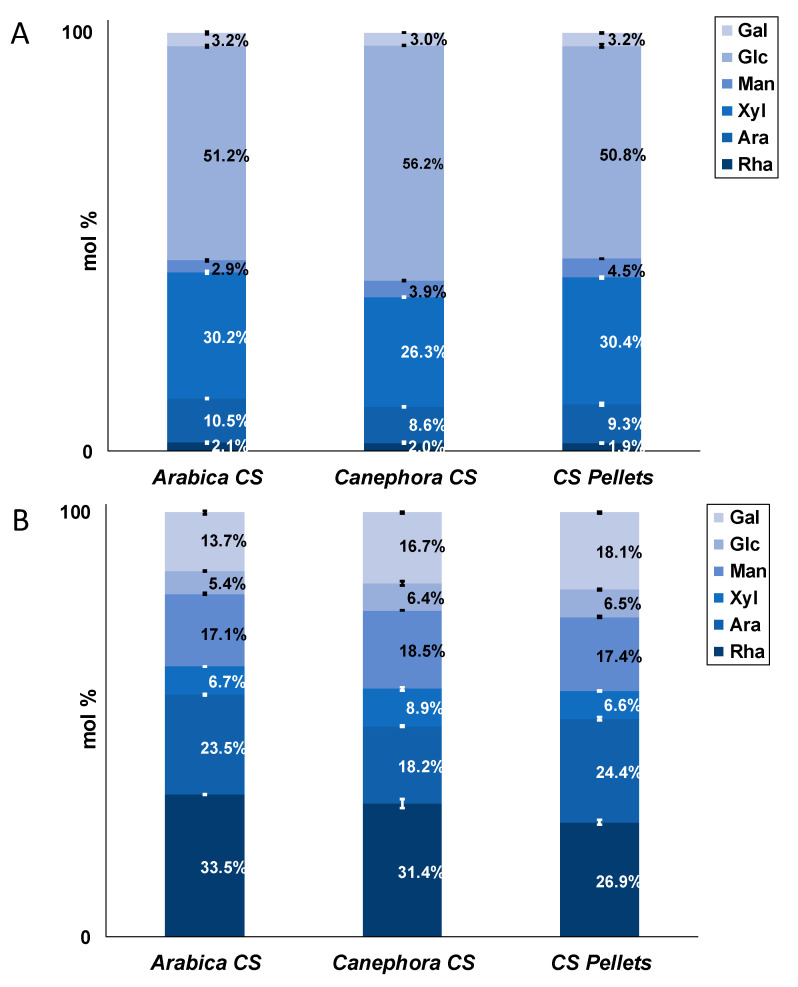
Neutral monosaccharide composition (in mol %) of insoluble dietary fiber (**A**) and soluble dietary fiber (**B**) polysaccharides from coffee silver skin from *Coffea arabica* (*arabica* CS) and *Coffea canephora* (*canephora* CS) as well as coffee silver skin pellets (CS pellets) (Gal, galactose, Glc, glucose; Man, mannose; Xyl, xylose; Ara, arabinose; Rha, rhamnose); n = 2.

Monosaccharide analysis after acid hydrolysis showed that glucose (50.8–56.2%) and xylose (26.3–30.4%) were the main monomers of IDF polysaccharides (Figure 2A). Although the monomer composition of IDF polysaccharides of *arabica* and *canephora* CS and CS pellets differ, these differences do not appear to be a large factor regarding both nutritional and technofunctional properties of CS. The high glucose content is mainly due to the cell wall polysaccharide cellulose (see below). Being a dicotyledonous plant, coffee cell walls also contain xyloglucans as demonstrated by Oosterveld et al. [46] potentially contributing to both glucose and xylose contents measured here. Uronic acids were detected at levels of 9.8 ± 0.3 g/100 g (*arabica* CS), 10.1 ± 0.2 g/100 g (*canephora* CS), and 8.6 ± 0.3 g/100 g (CS pellets). 

In SDF polysaccharides, rhamnose (26.9–33.5%), arabinose (18.2–24.4%), mannose (17.1–18.5%), and galactose (13.7–8.1%) were dominant monomers (Figure 2B). Again, differences among SDF of *arabica* and *canephora* CS as well as CS pellets were small (Figure 2B). Uronic acid contents of 35.9 ± 2.4 g/100 g (*arabica* CS), 30.0 ± 1.0 g/100 g (*canephora* CS), and 32.6 ± 2.1 g/100 g (CS pellets), respectively, indicate the presence of pectic polysaccharides. In combination with the high portion of rhamnose, rhamnogalacturonans appear to be dominant (see below). Other polysaccharides such as type II arabinogalactans, galactomannans, and glucomannans in coffee beans [47] are potential contributors to the arabinose, mannose, glucose, and galactose contents, too. 

#### 3.1.2. Methylation Analysis of Insoluble and Soluble Dietary Fiber Polysaccharides

Based on methylation analysis, 1,4-linked glucopyranose was identified as the main component (Table 3), demonstrating the dominance of cellulose in CS IDF. Although high portions of terminal xylose were analyzed, 1,4,6-linked glucopyranose units were not detected. Thus, although the presence of (at least small amounts of) xyloglucans is likely, we were not able to unambiguously demonstrate its existence in CS. Furthermore, the large portion of xylose detected in the monomer analysis (Section 3.1.1) also stems from xylans. The backbone of xylans consists of β-1,4-linked xylopyranose units, which were detected at levels between 14.2–17.3%. Xylans appear to be predominantly linear, being typical for xylans from secondary plant cell walls. Thus, methylation analysis of IDF indicates a large portion of secondary cell walls in CS. IDF methylation analysis data showed no distinct differences among *arabica* and *canephora* CS and CS pellets. 

Additionally, methylation analysis data of SDF showed no large differences between *arabica* and *canephora* CS. However, some minor PMAAs were not detected in CS pellets (Table 3). Monosaccharide analysis indicated the presence of rhamnogalacturonan. The backbone of type I rhamnogalacturonan consists of alternating α-1,2-linked-rhamnopyranose and α-1,4-linked galactopyranuronic acid units; in agreement with our assumption, methylation analysis demonstrated comparably large amounts of 1,2-linked rhamnose. Detected 1,2,4-rhamnopyranose units represent branching points, bearing neutral arabinan and (arabino)galactan side-chains. Thus, portions of 1,5-linked arabinose and 1,4-linked galactose units can be assigned to rhamnogalacturonan I side chains, with arabinans dominating over (arabino)galactans (Table 3). CS arabinans appear to be mainly linear, because branching sites in position *O*2/and or *O*3 were not identified in addition to 1,5-arabinofuranose backbone units. Portions of 1,3,6-galactopyranose and 1,6-galactopyranose units suggest type II arabinogalactans, which are common coffee polysaccharides [48,49,50]; however, only 1,6-galactopyranose units were detected in CS pellets (Table 3).

### 3.2. Lignin as Insoluble Dietary Fiber Constituent

**Figure 3:**
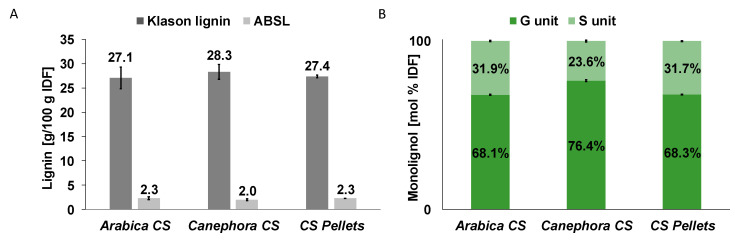
Insoluble dietary fiber (IDF) lignin contents according to Klason and acetyl bromide soluble lignin (ABSL) methods (**A**), and monolignol ratios liberated from IDF following thioacidolysis (**B**) of coffee silver skin from *Coffea arabica* (*arabica* CS) and *Coffea canephora* (*canephora* CS) and coffee silver skin pellets (CS pellets). S-unit, syringyl units; G-unit, guaiacyl units; n = 2.

Due to the comparably low specificity of the commonly used assays, quantitative analysis of lignin in low-lignified tissues is problematic [51,52] and often misleading. Here, Klason lignin values of 27.1–28.3 g/100 g IDF are distinctly higher than ABSL values of 2.0–2.3 g/100 g IDF (Figure 3A). However, Klason lignin and ABSL data, respectively, are comparable among *arabica* and *canephora* CS and CS pellets. Both Klason lignin and ABSL methods are non-specific, differing, however, in co-analyzed compounds [51]. In this specific matrix, Maillard reaction products such as melanoidins, which are not completely cleaved by hydrolysis, may largely contribute to Klason lignin contents. This observation was previously described by Valiente et al., who determined Klason lignin contents in green and roasted coffee beans [53]. 

Because of the lack of specificity, both applied “quantitative” methods do not confirm the existence of actual lignin (lignin according to its definition and botanical purpose [54]). Therefore, lignin was identified by analyzing lignin monomers following thioacidolysis (Figure 3B). In all CS, guaiacyl (G) and syringyl (S) units (G-units describe polymerized coniferyl alcohol, whereas S-units describe polymerized sinapyl alcohol) were detected, thereby proving lignin as IDF and therefore CS constituent. *p*-Hydroxyphenyl (H)-units, which describe polymerized *p*-coumaryl alcohol, were not detected. G units generally dominate over S units with G and S ratios being comparable between *arabica* CS and CS pellets, whereas *canephora* CS have a slightly smaller portion of S units and larger portion of G units. Because pellets are assumed to contain a large portion of *arabica* CS, a similarity of these with *arabica* CS can easily be explained. 

### 3.3. Protein and Amino Acid Composition

With values ranging from 17.8 ± 0.1 g/100 g to 22.2 ± 0.5 g/100 g, CS is rich in protein (Table 2). Ballesteros et al. [12] and Borrelli et al. [13] determined protein contents of 18.7 g/100 g and 18.6 g/100 g, respectively. However, the analytical procedure to determine protein contents differed slightly from the method used here, so that direct comparability is not possible. Nevertheless, the results indicate that despite natural variability, e.g., due to climatic effects, fertilization practices, and minor differences in determining these contents, CS can be considered as rich in protein. With regard to the use of CS as a food ingredient, high protein contents are desirable. However, besides the mere protein content, its amino acid composition is also important. The applied method for the determination of amino acids is limited in the sense that the determination of tryptophan is not possible, and the amino acids methionine and cysteine are potentially underestimated due to partial degradation. Therefore, these two amino acids were only analyzed qualitatively. The amino acid composition of the studied CS samples is largely comparable (Table 4). Glutamic acid, aspartic acid, and leucine are dominant. Note that glutamic acid and aspartic acid contents also include glutamine and asparagine as the amides are transformed into the acids during hydrolysis. The essential amino acids leucine, valine, phenylalanine, isoleucine, threonine, histidine, and lysine are present in considerable proportions (Table 4). The essential amino acid methionine was detected but not quantified (see above). Machado et al. determined free amino acids in a mixture of *arabica* and *canephora* CS and reported large amounts of glutamic acid and aspartic acid [55]. In addition, all essential amino acids, except methionine, were present in free form [55]. 

It has been suggested that arabinogalactans are present in the SDF fraction of CS. Type II arabinogalactans are usually associated with proteins, with arabinogalactan proteins consisting largely of the amino acids hydroxyproline, alanine, threonine, glycine, and serine. Although hydroxyproline was identified in considerable amounts, the mentioned amino acids do not dominate the amino acid composition of the proteins in CS SDF (Table 4). 

### 3.4. Fat and Fatty Acid Composition

**Figure 4:**
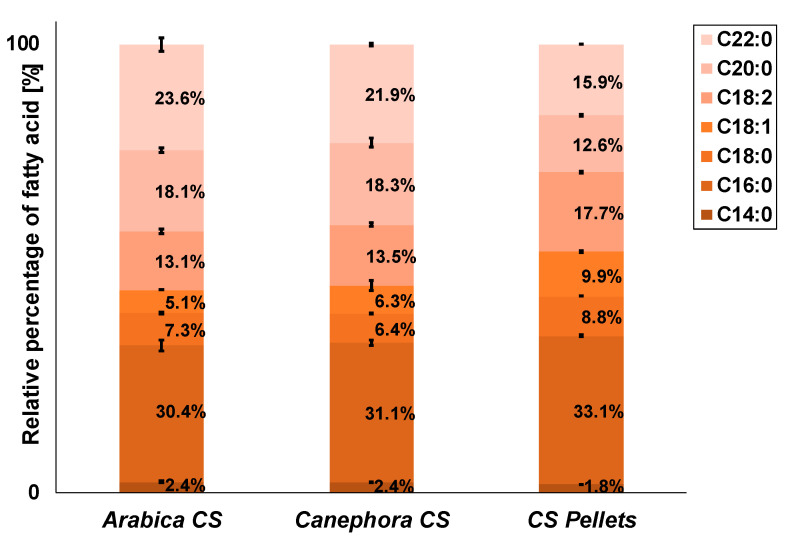
Fatty acid composition of lipids from coffee silver skin from *Coffea arabica* (*arabica* CS) and *Coffea canephora* (*canephora* CS) as well as coffee silver skin pellets (CS pellets); percent of total fatty acids. C14:0 myristic acid; C16:0 palmitic acid; C18:0 stearic acid; C18:1 oleic acid; C18:2 linoleic acid; C20:0 arachidic acid; C22:0 behemic acid; n = 2.

Low fat contents were observed for all CS (Table 2). *Canephora* CS have a slightly higher fat content (1.82 ± 0.04 g/100 g) as compared to *arabica* CS (1.57 ± 0.08 g/100 g) and CS pellets (1.50 ± 0.02 g/100 g). This corroborates data published by Iriondo-DeHond et al. reporting a higher fat content for *canephora* CS as compared to *arabica* CS, too [3]. The fatty acid composition is largely comparable among the different CS, with slight deviations for the pellets (Figure 4). Lauric acid (C12:0) was generally present at levels of about 0.1% (not shown in Figure 4). Saturated fatty acids dominate the fatty acid composition adding up to approximately 80%. Among these, palmitic acid (C16:0) is the main fatty acid, ranging from 30.4% to 33.1% (Figure 4). In addition, notable portions of behenic acid (C22:0) and arachidic (C20:0) acid are present in CS (Figure 4). The unsaturated fatty acid fraction is mainly composed of oleic acid (C18:1) and linoleic acid (C18:2). Our data are in accordance with data from Toschi et al., who also identified palmitic acid, behenic acid, and arachidic acid as the main saturated fatty acids [23]. Bessada et al. also showed a similar fatty acid profile in lipids from *canephora* CS of different geographical origins [22]. In addition to comparable portions of oleic acid and linoleic acid, however, a small portion of α-linoleic acid was also reported Toschi et al. and Bessada et al. [22,23].

### 3.5. Ash Contents and Mineral Composition

Ash contents of CS are quite high with values ranging from 8.15 to 11.24 g/100 g (Table 2) indicating a large mineral content. Therefore, semiquantitative elemental screening was performed (Table 5). Data on minerals were comparable for *arabica* and *canephora* CS as well as CS pellets, except for copper, which occurred in higher amounts in *canephora* CS (Table 5). Calcium, potassium, and magnesium were the most abundant mineral elements. As these belong to the macro elements, high amounts are to be expected. Several studies reported potassium, magnesium, and calcium as the highest concentrated elements in roasted coffee beans [56,57,58,59]. Ballesteros et al. reported potassium as the most abundant element in a mixture of *arabica* and *canephora* CS, followed by magnesium [12]. Costa et al. determined the macrominerals in CS, with potassium, magnesium, and calcium being the main ones [27].

The trace elements iron, manganese, copper, chromium, cobalt, and molybdenum were detected in all CS samples. They are essential to metabolic functions of the human body [60]. However, elements such as cadmium, mercury, lead, and arsenic have no known beneficial effects and may even be toxic [61]. The FAO/WHO has set a provisional tolerable weekly intake (PTWI) for lead of 25 µg/kg body weight and for cadmium of 7 µg/kg body weight [62]. According to the European Food Safety Authority (EFSA), the PTWI for lead was no longer appropriate and since then, no new value has been set and the major goal is to keep the lead content as low as possible. However, this PTWI can be used here as a guide for toxicological assessment as there are no other guideline values for lead available at this time. Arsenic and mercury were not identified in any of the CS in this study. Cadmium was measured in *arabica* and *canephora* CS with values ranging from 0.075–0.10 mg/kg. In CS pellets, cadmium was detected but only with small intensities. Lead was determined to range from 0.16–0.65 mg/kg in the CS, with *arabica* CS having the highest and CS pellets having the lowest value (Table 5). A human being with 70 kg of weight can ingest 490 µg of cadmium and 1750 µg of lead before reaching the PTWI. For example, 4.9 kg or 2.6 kg of CS, respectively, could be ingested weekly to reach the PTWI for cadmium or lead. Because the lead content should be kept as low as possible, less CS should be ingested in this respect. However, consumption of CS in these amounts appear to be highly unlikely. For example, Pourfarzad et al. used CS as an ingredient in barbari flat bread with an amount of 5 g/100 g flour [8]. For bread, levels of 0.028 mg/kg were found for cadmium [63]. Because CS are added in low levels to food products, it can be assumed that they do not contribute decisively to cadmium and lead contents in the final food product. However, it should be noted that CS as possible additives should have a very low content of cadmium and lead, regardless of the food to which they are added. This applies in particular to lead, as the toxicity of this chemical element has not yet been conclusively clarified.

Of the elements studied, gallium, bromine, antimony, iodine, samarium, europium, gadolinium, dysprosium, erbium, ytterbium, and uranium were only measured with low intensities (data not shown).

Mineral contents are highly influenced by soil characteristics, such as content of organic matter and/or pH value [64]. Additionally, the application of chemical products such as fertilizers, fungicides, insecticides, and herbicides may affect element levels, as these products potentially contain multiple metals [64]. Here, only *canephora* CS from India and *arabica* CS from Brazil, India, Mexico, and El Salvador were analyzed. In order to obtain a more representative view on CS minerals, CS from other countries should be investigated.

### 3.6. Acrylamide, HMF, Furfural Alcohol, and Caffeine

Acrylamide was determined in CS samples with a content of 152 µg/kg (*arabica* CS), 161 µg/kg (*canephora* CS), and 24.0 µg/kg (CS pellets) (Table 2). Because acrylamide is a process contaminant formed during coffee roasting with asparagine and sucrose being the main precursors [65], its presence was expected. Iriondo-DeHond et al. determined acrylamide in a CS extract (489 µg/kg CS extract) and in the insoluble fraction of CS [3]. The acrylamide content in coffee beans is influenced by the degree of roasting. Studies by Soares et al. and Lachenmeier et al. showed that light roasted coffee beans had a higher acrylamide content than dark roasted beans [43,65]. It can be assumed that the influence of the degree of roasting on the acrylamide content also applies to CS, which makes it difficult to compare the determined contents with those reported in other studies. In the European Union, benchmark levels for acrylamide contents in food products were defined in commission regulation (EU) 2017/2158. For roasted coffee, the benchmark level is 400 µg/kg and for wheat-based bread, 50 µg/kg [66]. With a maximum level of 161 µg/kg, the acrylamide content of CS samples is below the guideline values for roasted coffee. Again, Pourfarzad et al. used CS as an ingredient in barbari flat bread (5 g CS/100 g flour) [8]. Thus, for 1 kg flour, 50 g of CS would be used. Accordingly, 50 g of CS would contain 1.2–8.1 µg of acrylamide, which would not contribute mainly to the exceedance of the benchmark level value of 50 µg/kg. Additionally, Garcia-Serna et al. determined the acrylamide content of CS enriched biscuits and did not detect any significant difference in acrylamide contents between the biscuits with and without added CS [9].

In addition to acrylamide, other heat induced contaminants such as furfuryl alcohol and HMF were analyzed in CS. Furfuryl alcohol has been classified by IARC as possibly carcinogenic to humans (Group 2B) [25]. HMF has not yet been classified by IARC, but studies reported carcinogenic activity of HMF in animal experiments [67]. Furfuryl alcohol was not detected with the analytical method used here. HMF was detected in *arabica* and *canephora* CS, but the content was below the limit of quantification of 22.9 mg/kg. In CS pellets, HMF was not identified. HMF contents in *arabica* and *canephora* CS have already been described earlier ranging from 56.8 mg/kg to 3210 mg/kg [22,27,28]. However, the methods described in the literature differ substantially from the method used here, which may explain the differences of the values compared to those in this study [22,27,28]. Moreover, HMF is formed from sugars during the coffee roasting process and the content of HMF therefore depends on the roasting profile [68]. The EFSA recommended a benchmark dose (BMD) of 29.5 mg/kg/d based on a study by the National Toxicology Program (NTP) [69]. With HMF levels of less than 22.9 mg/kg in CS, CS are considered to be of low concern in this regard.

Interestingly, the method used to detect furfuryl alcohol and HMF was also able to detect 16-*O*-methylcafestol and kahweol, which are typical markers used to distinguish roasted beans of *Coffea arabica* and *Coffea canephora* [70]. Therefore, we detected 16-*O*-methylcafestol in *canephora* CS and quantified kahweol in *arabica* and *canephora* CS and CS pellets (data not shown). Levels of kahweol were lower in *canephora* CS when compared to *arabica* CS or CS pellets. Thus, discrimination between CS from species of *Coffea arabica* and *Coffea canephora* based on 16-*O*-methylcafestol and kahweol would also be possible.

The contents of caffeine in *arabica* CS (0.80 ± 0.002 g/100 g) and CS pellets (0.76 ± 0.01 g/100 g) were comparable whereas *canephora* CS had higher values (0.86 ± 0.03 g/100 g). The caffeine content in coffee is approximately 1–2 g/100 g with the values being influenced by the degree of roasting and species [71]. Additionally, *Coffea* *canephora* coffee beans were found to contain more caffeine than *Coffea arabica*, with caffeine contents decreasing with increasing degree of roast [70,71]. Thus, the caffeine contents of CS are lower as compared to coffee beans. However, levels of caffeine of 0.6–1.2 g/100 g were reported for *canephora* CS and 0.4 g/100 g up to 1.0 g/100 g for *arabica* CS [18,22,28]. The presence of caffeine in CS opens up new applications, such as the addition of CS as a natural ingredient to enrich food products with caffeine. Since CS are produced in a large amount as a by-product, it appears also possible to extract caffeine from CS. However, in addition to stimulating the central nervous system, caffeine has been linked to cardiovascular problems when consumed in excess amounts [72]. In its risk assessment, EFSA concluded that a single dose about 3 mg/kg body weight could be assumed to be safe [72]. Thus, a person with 70 kg of body weight can ingest a single dose of 210 mg caffeine. To reach this dose, approximately 30 g of CS need to be ingested. Pourfarzad et al. used 5 g CS/100 g flour to make a barbari flat bread [8]. Thus, six barbari flat breads need to be ingested at one time to achieve this single dose. Bertolino et al. also used a maximum of 6.3 g CS per 100 g yogurt [10]. Consuming four yogurts at once would result in a dose of 190 mg caffeine. Therefore, CS are considered safe in terms of caffeine content. However, consumption of caffeine is not recommended for children and pregnant women [72]. These considerations should be taken into account for a possible use of CS in food.

## 4. Conclusions

Based on our research, CS appear to be suitable for use in the food industry; however, detailed safety considerations need to reflect the actual application. High (soluble) fiber contents, high protein contents, and low fat contents appear to be beneficial for food applications. Heat-induced contaminants were found in comparably low concentrations only. Mycotoxins were not investigated in this study, but studies of Iriondo-DeHond showed that CS can be considered as a low risk in this respect, too [3]. No significant differences were identified between CS of the species *Coffea arabica* and *Coffea canephora* as well as CS pellets, making all of them equally suitable for use in the food industry. However, the novel food status of CS in the European Union currently appears unclear. Klingel et al. [5] suggested a consultation procedure with the responsible authority to determine whether a novel food approval is necessary prior to the application of CS as food ingredient.

## Figures and Tables

**Table 1:** Validation parameters for the quantitative determination of furfuryl alcohol and 5-hydroxymethylfurfural (HMF) using nuclear magnetic resonance spectroscopy Adapted from Ref. [44].

Validation Parameter	Furfuryl Alcohol	HMF
Detection limit (mg/kg)	11.6	6.3
Quantification limit (mg/kg)	39.4	22.9
Recovery (%)	93–97	101–107
Precision (%)	5.8–6.1	6.9–8.3
Linearity range (mg/kg)	7.5–5625	7.5–5625

**Table 2:** Chemical composition of coffee silver skin from *Coffea arabica* (*arabica* CS) and *Coffea canephora* (*canephora* CS) as well as coffee silver skin pellets (CS pellets) based on dry material.

Constituent	*Arabica* CS	*Canephora* CS	CS Pellets
Dietary Fiber (g/100 g)	67.0 ± 1.0	62.0 ± 0.4	59.1 ± 0.02
Insoluble (g/100 g)	56.0 ± 0.4	53.2 ± 0.3	46.0 ± 0.2
Soluble (g/100 g)	11.0 ± 1.7	8.8 ± 0.5	13.1 ± 0.2
Fat (g/100 g)	1.57 ± 0.03	1.50 ± 0.02	1.82 ± 0.04
Ash (g/100 g)	8.15 ± 0.08	9.50 ± 0.14	11.24 ± 0.01
Protein (g/100 g)	18.1 ± 0.2	22.2 ± 0.5	17.8 ± 0.1
Caffeine (g/100 g)	0.80 ± 0.002	0.86 ± 0.03	0.76 ± 0.01
Acrylamide (µg/kg)	152	161	24.0
HMF	pos. (<LOQ)	pos. (<LOQ)	pos. (<LOQ)
Furfuryl alcohol	n.d.	n.d.	n.d.
Moisture content (%)	6.15 ± 0.12	6.57 ± 0.06	7.64 ± 0.02

LOQ, limit of quantification; n.d., not detectable; n = 3 for caffeine; n = 2 for dietary fiber, fat, ash, protein, HMF, and furfuryl alcohol; n = 1 for acrylamide; relative standard deviation 15% for acrylamide.

**Table 3:** Glycosidic linkage composition of insoluble (IDF) and soluble (SDF) dietary fiber polysaccharides from coffee silver skin from *Coffea arabica* (*arabica* CS) and *Coffea canephora* (*canephora* CS) as well as coffee silver skin pellets (CS pellets).

	IDF [%]			SDF [%]		
Glycosidic Linkage	*Arabica* CS	*Canephora* CS	CS Pellets	*Arabica* CS	*Canephora* CS	CS Pellets
t-Arabinofuranose	4.6 ± 0.04	3.6 ± 0.2	5.2 ± 0.5	22.6 ± 1.1	16.8 ± 0.03	23.0 ± 0.3
1,5-Arabinofuranose				14.8 ± 1.2	11.8 ± 2.2	14.6 ± 0.02
t-Galactopyranose				7.0 ± 0.8	7.0 ± 1.0	7.5 ± 1.2
1,4-Galactopyranose				3.7 ± 0.7	5.3 ± 0.6	6.5 ± 0.9
1,6-Galactopyranose				5.4 ± 0.3	6.1 ± 0.3	6.0 ± 0.2
1,3,6-Galactopyranose				2.4 ± 0.4	3.5 ± 0.2	n.d.
t-Glucopyranose				1.7 ± 0.3	2.9 ± 0.5	n.d.
1,4-Glucopyranose	60.5 ± 1.3	68.5 ± 0.3	57.7 ± 5.4	7.0 ± 0.4	10.6 ± 0.8	6.2 ± 0.1
t-Mannopyranose				2.8 ± 0.3	2.9 ± 0.5	n.d.
1,2-Rhamnopyranose				14.9 ± 2.2	11.9 ± 0.6	12.2 ± 0.8
1,2,4-Rhamnopyranose				6.5 ± 0.5	8.0 ± 2.1	10.3 ± 1.0
t-Xylopyranose	16.9 ± 1.8	14.2 ± 2.0	21.2 ± 3.9	11.2 ± 3.7	13.3 ± 2.4	13.8 ± 2.3
1,4-Xylopyranose	17.9 ± 0.5	13.7 ± 1.9	15.9 ± 1.0	n.d.	n.d.	n.d.

t = terminal; n.d., not detected; n = 2.

**Table 4:** Amino acid composition of proteins from coffee silver skin from *Coffea arabica* (*arabica* CS) and *Coffea canephora* (*canephora* CS) as well as coffee silver skin pellets (CS pellets); percent of total amino acids.

	*Arabica* CS (%)	*Canephora* CS (%)	CS Pellets (%)
Glutamic acid	13.79 ± 0.16	13.60 ± 0.22	14.68 ± 0.07
Aspartic acid	9.86 ± 0.46	9.79 ± 0.16	9.73 ± 0.10
Leucine	9.79 ± 0.02	9.18 ± 0.11	9.31 ± 0.02
Valine	7.46 ± 0.01	7.06 ± 0.08	7.22 ± 0.03
Phenylalanine	6.89 ± 0.02	6.87 ± 0.01	6.60 ± 0.03
Glycine	6.87 ± 0.04	6.30 ± 0.31	6.64 ± 0.07
Isoleucine	6.95 ± 0.01	6.76 ± 0.09	6.61 ± 0.03
Proline	6.65 ± 0.08	6.88 ± 0.10	6.70 ± 0.09
Alanine	5.64 ± 0.06	5.72 ± 0.12	5.69 ± 0.05
Serine	5.18 ± 0.03	4.45 ± 0.07	4.95 ± 0.03
Hydroxyproline	4.86 ± 0.07	3.59 ± 0.06	3.89 ± 0.01
Threonine	4.62 ± 0.02	4.22 ± 0.04	4.42 ± 0.01
Histidine	4.47 ± 0.01	4.11 ± 0.17	4.48 ± 0.05
Lysine	3.95 ± 0.05	3.49 ± 0.10	3.28 ± 0.09
Tyrosine	3.01 ± 0.02	3.38 ± 0.43	3.68 ± 0.24
Arginine	pos. (<LOQ)	2.59 ± 0.09	pos. (<LOQ)
Cysteine	pos. (<LOQ)	pos. (<LOQ)	pos. (<LOQ)
Methionine	pos. (<LOQ)	pos. (<LOQ)	pos. (<LOQ)

LOQ, limit of quantification; LOQ for arginine: 0.4 µmol/L; LOQ for cysteine: 0.4 µmol/L; LOQ for methionine: 0.05 µmol/L; n = 3.

**Table 5:** Mineral composition of *Coffea* *arabica* silver skin (*arabica* CS) and *Coffea canephora* silver skin (*canephora* CS) as well as coffee silver skin pellets (CS pellets).

Elements	*Arabica* CS (mg/kg)	*Canephora* CS (mg/kg)	CS Pellets (mg/kg)
Calcium Ca	>10,000 *	>10,000 *	~10,000 *
Potassium K	~10,000 *	~20,000 *	>22,000 *
Magnesium Mg	>2000 *	>4000 *	>5000 *
Iron Fe	~1000 *	>600 *	~500 *
Silicium Si	650	560	580 *
Aluminium Al	215	155	425 *
Sodium Na	200	360	83
Manganese Mn	145	43	~65 *
Barium Ba	~130	73	~65 *
Copper Cu	98	185	70
Strontium Sr	68	38	68 *
Boron B	33	30	23
Titanium Ti	30	20	60
Zink Zn	25	33	12
Rubidium Rb	10	18	~42 *
Tin Sn	7.5	13	0.10
Chromium Cr	4.0	2.9	2.5
Nickel Ni	1.9	2.3	0.95
Lead Pb	0.75	0.65	0.16
Cobalt Co	0.60	0.95	0.85
Vanadium V	0.45	0.30	1.1
Zirconium Zr	0.31	0.18	0.38
Molybdenum Mo	0.26	0.21	0.13
Cerium Ce	0.21	0.18	0.85
Lanthanum La	0.15	0.15	0.45
Scandium Sc	0.18	0.15	0.33
Yttrium Y	0.16	0.10	0.17
Neodymium Nd	0.11	0.10	0.31
Caesium Cs	0.085	0.035	0.17
Gallium Ga	0.075	n.d.	0.18
Cadmium Cd	0.075	0.10	n.d.
Silver Ag	n.d.	0.065	n.d.
Niobium Nb	n.d.	n.d.	0.12
Praseodymium Pr	n.d.	n.d.	0.10–0.11

* High count rates (>1E6 cps); values serve only as an orientation; n.d., not detected; n = 3 for *arabica* CS and *canephora* CS; n = 2 for pellets; measurement uncertainty of 50% is to be assumed.

## Data Availability

The data presented in this study are available on request from the corresponding author.
